# QuickStats

**Published:** 2013-03-29

**Authors:** Li-Hui Chen, Holly Hedegaard, Margaret Warner

**Figure:**
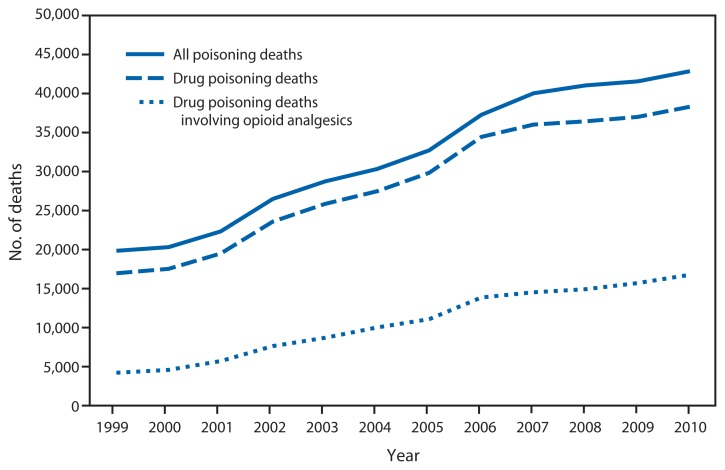
Number of Deaths From Poisoning,^*^ Drug Poisoning,^†^ and Drug Poisoning Involving Opioid Analgesics^§^ — United States, 1999–2010 ^*^Poisoning deaths include those resulting from drugs, and those associated with solid or liquid biologics, gases or vapors, or other substances. Poisoning deaths are from all manners, including unintentional, suicide, homicide, and undetermined intent. ^†^Drug poisoning deaths include unintentional or intentional poisoning deaths resulting from overdoses of a drug, being given the wrong drug, taking the drug in error, or taking a drug inadvertently. ^§^Among deaths with drug poisoning as the underlying cause, the *International Classification of Diseases, 10th Revision* codes T40.2–T40.4 were used to indicate whether opioid analgesics were involved.

From 1999 to 2010, the number of U.S. drug poisoning deaths involving any opioid analgesic (e.g., oxycodone, methadone, or hydrocodone) more than quadrupled, from 4,030 to 16,651, accounting for 43% of the 38,329 drug poisoning deaths and 39% of the 42,917 total poisoning deaths in 2010. In 1999, opioid analgesics were involved in 24% of the 16,849 drug poisoning deaths and 20% of the 19,741 total poisoning deaths.

**Sources:** National Vital Statistics System. Mortality data. Available at http://www.cdc.gov/nchs/deaths.htm.

Warner M, Chen LH, Makuc DM, Anderson RN, Miniño AM. Drug poisoning deaths in the United States, 1980–2008. NCHS data brief, no 81. Hyattsville, MD: US Department of Health and Human Services, CDC, National Center for Health Statistics; 2011. Available at http://www.cdc.gov/nchs/data/databriefs/db81.pdf.

